# Potential Cross-Talk between Alternative and Classical NF-κB Pathways in Prostate Cancer Tissues as Measured by a Multi-Staining Immunofluorescence Co-Localization Assay

**DOI:** 10.1371/journal.pone.0131024

**Published:** 2015-07-17

**Authors:** Ingrid Labouba, Cécile Le Page, Laudine Communal, Torbjoern Kristessen, Xiaotian You, Benjamin Péant, Véronique Barrès, Philippe O. Gannon, Anne-Marie Mes-Masson, Fred Saad

**Affiliations:** 1 Institut du cancer de Montréal / Centre de recherche du Centre hospitalier de l’Université de Montréal (CRCHUM), 900 rue St-Denis, Montreal, Canada; 2 Visiopharm, Argen Allé 3, Hoersholm, Denmark; 3 Department of Medicine, Université de Montréal, Montreal, Canada; 4 Division of Urology, CHUM and Department of Surgery, Université de Montréal, Montreal, Canada; University of Kentucky College of Medicine, UNITED STATES

## Abstract

**Background:**

While the classical NF-κB/p65 pathway is known to be involved in prostate cancer progression and is associated with poor patient outcome, the role of the NF-κB /RelB alternative protein is not well defined. Here we analyzed the activation of both NF-κB pathways in prostate cancer tissues and correlate this activation with clinical features of the disease.

**Methods:**

A multiple immunofluorescence technique was employed to concomitantly and quantitatively visualize the nuclear localization of p65 and RelB in 200 paraffin embedded samples. Epithelia were defined using appropriate fluorochrome markers and the resulting immunofluorescent signals were quantified with an automated scoring system.

**Results:**

The nuclear frequency of p65 was found to be significantly increased in tumor tissues as compared with normal adjacent tissue, whereas the frequency for RelB was decreased (p < 0.001, Wilcoxon test). As previously reported, p65 nuclear frequency was associated with a risk of biochemical recurrence. Although, RelB nuclear frequency alone did not predict recurrence, the presence of activated RelB reduced the risk of recurrence associated with the activation of p65.

**Conclusion:**

For the first time p65/RelB co-distribution was assessed in prostate cancer tissues and suggested a negative crosstalk between the two NF-κB pathways in prostate cancer progression.

## Introduction

Prostate cancer is the most frequently diagnosed cancer in North American men and is the second leading cause of cancer-related death in males after lung and colon cancers [1]. Typically, prostate cancer remains localized within the prostate gland. However, it may also grows aggressively beyond the prostate capsule and lead to metastasis formation [2]. While some prostate cancer patients only require active monitoring of the disease, others need more radical treatments [3, 4]. At present, the majority of patients with localized prostate cancer undergo surgery, radiotherapy or androgen deprivation therapy as first line treatment [5]. However, despite the high success rates obtained with these treatments, 25% of patients develop biochemical recurrence (BCR) after hormone-therapy treatment and progress to a more aggressive disease [6, 7]. Thus, a major challenge in prostate cancer is the proper discrimination of patients with a latent or slow progressing disease from those with a more aggressive form of cancer. Such discrimination will help tailor treatment appropriately for individual patients. To better stratify prostate cancer patients prognosis, the identification of specific molecular biomarkers able to predict outcome would represent an important advancement over existing clinical tools. In this context, our group has been the first to address the prognostic potential of the p65 subunit of Nuclear Factor (NF)-κB in prostate cancer [8] and highlight a potential role of Nuclear Factor (NF)-κB in prostate cancer progression [9, 10].

The NF-κB family includes five transcription factor proteins characterized by their Rel-homology domain. There are subdivided into two main groups: Class I (NFκB1 and NFκB2) and class II (RelA/p65, RelB, and c-Rel). The NFκB1 and NF-κB2 are translated in the p100 and p105 proteins that are processed in their respective p50 and p52 subunits [11]. Two major pathways control NF-κB signaling: the classical pathway, in which the heterodimer p65/p50 is the main active dimer, and the alternative pathway where the RelB/p52 is the active dimer. Under normal conditions, NF-κB is retained inactive in the cytosol by the IκB or p100 proteins. During the activation, in the classical pathway (IKKβ-dependent), the IKK complex phosphorylates IκB, inducing its ubiquitination and degradation by the proteasome. This leads to the nuclear translocation of the p65/p50 or p50/c-rel dimers. In the alternative pathway (IKKα-dependent), the IKK complex regulates the processing of the p100 precursor, as opposed to IκB [12–15], which generates p52 and the nuclear translocation of the p52/RelB dimer.

Numerous molecular and biological functions, such as inflammation [16, 17], angiogenesis [18], survival, migration and invasion [19] have been shown to be associated with the activity of the classical NF-κB nuclear factors in cancer progression (reviewed in [20]). While significant work has focused on the study of the classical NF-κB pathway, recently, attention has been directed towards the alternative NF-κB pathway, and particularly in regards to its influence on inflammation. The biological functions of NF-κB also involve crosstalk between the classical and alternative pathways at different levels, from upstream signaling to nuclear interactions via the formation of diverse NF-κB dimers. Depending on the context and stimuli, the two pathways can cooperate, or interfere negatively, in the regulation of gene expression. Furthermore, the biological functions directed by the crosstalk of both pathways remain poorly understood and has never been investigated in prostate cancer cells.

In prostate cancer, several studies have reported that both RelB and p65 may be putative prognostic biomarkers associated with disease progression. We, and others, have previously observed by immunohistochemistry (IHC) in prostate cancer tissue, that elevated amounts of nuclear p65 were associated with more aggressive disease [8, 21]. Subsequently, it was shown that the nuclear localization of p65 is highly predictive of lymph node invasion [9] and was associated with biochemical recurrence (BCR), either alone or in combination with activated ErbB/Akt signaling [21–25]. More recently, we observed an increased presence of nuclear RelB in prostate cancer tissues compared to non-neoplastic tissues, thereby suggesting that the alternative NF-κB pathway is also activated during the course of disease progression [10]. In addition, it was shown in an *in vitro* model that the inhibition of RelB expression increases the proliferation of 22Rv1 castrate-resistant prostate cancer cells and stimulates cellular autophagy [26]. Other studies found that RelB increases radiosensitivity of the castrate-resistant cells [27–30] seemingly by up-regulating IL-8 [31]. In line with these first observations, it was also shown that RelB is overexpressed in prostate glands in response to androgen deprivation [32] and radiation treatment [33], and also induces expression of of β-galactoside α2,3-sialyltransferase, a gene involved in the gangliosides expression and cancer progression [34]. More recently, a negative crosstalk was shown between RelB and AR, and association with survival and metastatic cancer [35].

In order to estimate the role of RelB and the crosstalk between the classical and the alternative NF-κB pathways, we implemented a multi-staining fluorescence technique so as to quantitatively analyze the simultaneous presence of nuclear p65 and RelB in the same prostate cancer tissue cores. The quantitation of the fluorescent signal was supported by an automated system. To evaluate the biological role of both NF-κB pathway activities, we correlated the nuclear localization of p65 or RelB with clinical parameters and risk of biochemical recurrence to better undrstand the potential role of the NF-κB pathway crosstalk in prostate cancer progression.

## Materials and Methods

### Cohort of patients

The current study was based on a retrospective cohort of 200 prostate cancer patients whose Formalin Fixed Paraffin Embedded (FFPE) primary prostate cancer specimens, were used to construct the Tissue Microarrays (TMAs). All patients underwent surgery between 1992 and 2006 and provided informed written consent. All consents were securely filed in a locked place and scanned consents in a password protected electronic folder. The inclusion criterion for this retrospective cohort study was the absence of any treatment before the Radical prostatectomy (RP). After a screening review of the clinical data, 11 patients were excluded from the study, as they did not meet the inclusion criterion. The average patient follow-up was 96 months. BCR (Biochemical Recurrence) was defined based on a PSA (Prostate Specific Antigen) relapse above 0.3 ng.ml^−1^ after date of surgery (RP). Recurrence-free interval was defined as the time between RP and the date of first PSA increase above 0.3 ng.ml^−1^. The final staging, grading and histological diagnosis was based on the clinical pathology report from the Hôpital Notre-Dame, (CHUM, Montreal, QC, Canada). Ethics approval was obtained from the appropriate review board (Comité d’éthique de la recherche du CHUM). Written consents were securely filed in a locked place, and electronically scanned consents were saved in a password-protected electronic folder. Ethics approval was obtained from the appropriate review board (Comité d’éthique de la recherche du CHUM). The main clinical parameters of the 189 cases of the cohort are: Mean follow-up, 95 months; 49% Gleason score >7; 44% Gleason score = 7; 7% Gleason score <7; 18 cases with seminal vesicles involvement against 170 without; 26% with extraprostatic extension, 32% with positive surgical margin; 3% with lymph node invasion; 142 with pathological stage 2 and 47 with pathological stage 3; the cancer specific rate is 3% and the biochemical recurrence is 28%.

### Tissue microarrays

From FFPE human tissue samples, tumor areas were selected based on reviews of Hematoxylin/Eosin (H&E)-stained slides by a pathologist. FFPE tumor blocks were then biopsied using a 0.6 mm diameter tissue arrayer needle and the resultant cores were arrayed onto a grid in a recipient paraffin block. Each TMA set contained, one core of a tumor sample and one core of a normal adjacent prostatic glandular tissue for a total of 100 patients. These TMAs were built in duplicate to analysis two independent cores of each tissue type per cases. TMA samples were sectioned, stained by both H&E-and immunohistochemistry (IHC) for High Molecular weight Cytokeratin (HMCK), and then subsequently underwent an independent pathology review to confirm and annotate the histology of each core.

### Immunohistochemistry (IHC)

IHC was performed using a Benchmark XT automated stainer (Ventana Medical System Inc. VMSI), Tucson, USA). Antigen retrieval was carried out with the Ventana Cell Conditioning 1 reagent (VMSI #950–124). The primary antibodies used were: anti-p65 (sc-8008), anti-RelB (sc-226), anti-cytokeratin (CK) 18 (sc-6259) and anti-PSA (sc-7638), all obtained from Santa Cruz Biotechnology Inc. (Santa Cruz, CA) and anti-CK19 (ms-198-P0) from Thermo Scientific Lab Vision (Ottawa, ON, Canada). The specificity of the above antibodies against p65 and RelB was previously verified by western-blot [26, 36]. Antibodies were diluted from 1:25 to 1:1000 in an antibody diluent buffer (VMSI #ADB250), except for anti-PSA that was diluted in phosphate buffered saline (PBS). Diluted antibodies were manually dispensed on the slides and incubated at 37°C for 60 min. Reactions were carried out using the UltraView DAB detection kit (VMSI #760–500) and slides were then counterstained with hematoxylin and bluing reagent (VMSI #760–2021 and #760–2037). All sections were scanned with a 20x 0.75NA objective with a resolution of 0.3225 mm, using the VS-110 slide scanner (Olympus, Richmond Hill, ON, Canada) and analyzed offside by side with HW cytokeratin staining to account for benign glands.

### Immunofluorescence (IF)

We produced an epithelial mask using several specific epithelial antigens to distinguish stromal and epithelial components within tissue. To ensure coverage of all prostate cancer cells, even in their most undifferentiated state, we used a cocktail of CK18, CK19 and PSA that were all labeled to emit in the orange channel. Slides were also stained with DAPI (blue) to identify nuclei. Secondary antibodies against p65 and RelB were labeled to emit in the red and green channels, respectively. This allowed us to distinguish specific staining of p65 and RelB in the nucleus and cytoplasm of epithelial cells.

Specifically, antigen retrieval was first carried out with Cell Conditioning 1 (VMSI; #950–124) using the Bench Mark XT automated stainer (Ventana Medical System Inc.). The primary antibodies against p65 and RelB were diluted 1:125 in PBS, manually applied to slides, and incubated at 37°C for 60 min. The following steps were manually done on the bench under conditions to protect slides from light. Both secondary fluorescent antibodies were incubated simultaneously for 45 min at room temperature (RT): anti-mouse Cy5 (#A10524, Life Technologies Inc., ON, CANADA) for p65 and anti-rabbit Alexa Fluor 488 (A488) (#A11008, Life Technologies Inc.) for RelB, were both diluted 1:250 in 1X PBS. After two successive washes with 1X PBS, TMA slides were blocked for 60 min with Mouse-On-Mouse blocking reagent (1 drop in 250 μL PBS, MKB-2213, Vector Laboratories, CA, USA) and then incubated for 90 min at RT with anti-PSA (1:100 in PBS). The TMA slides were washed twice and incubated for 45 min at RT with a secondary fluorescent anti-goat Cy3 (#705-165-003, Jackson ImmunoResearch Laboratories Inc., PA, USA) diluted at 1:250 in 1X PBS. Slides were then blocked once again with Mouse-On-Mouse blocking reagent overnight at 4°C. Subsequently, they were incubated for 90 min at RT with a mix of anti-CK18 and anti-CK19 (both at 1:100 in 1X PBS) and then 45 min at RT with secondary fluorescent anti-mouse Alexa Fluor 546 (A546 antibody) (#A10036, Life Technologies Inc.). Finally, slides were incubated for 15 min at RT with a 0.1% (w/v) solution of Sudan Black in 70% ethanol to quench the tissue auto-fluorescence. The slides were mounted with ProLong Gold Antifade Mountant with DAPI (P-36931, Life Technologies Inc.) for nuclei staining. Between each step, TMA slides were washed twice with 1X PBS. Slides were stored at 4°C and scanned the following day. A negative control TMA slide was also done in parallel and incubated with 1X PBS in place of all primary antibodies.

### Staining quantification

The staining and scoring were performed blinded to the study end-point. The tissue sections were scanned with an Olympus microscope and VS110 slide scanner linked to an OlyVia image viewer software (xvViewer.exe). Fluorescent staining was then quantified with the VisiomorphDP software (Visiopharm, Denmark) allowing for an automated image analysis. The staining via CK18/CK19/PSA markers was used to limit the analysis to epithelial areas as the region of interest #1 (ROI #1), while DAPI served to assess the fluorescence only in nuclei (ROI #2) (S1 Fig). Cores with defined ROI #1 and #2 were manually reviewed to ensure proper selection of epithelial cells and to remove surrounding tissue with necrosis or inflammatory zones from the ROI. Continuous values of fluorescence intensity for both ROI #1 and #2 were then collected by the VisiomorphDP software and transferred to Excel to define epithelial and nuclear NF-κB scores. The positive and negative signals were based on designated threshold values (mean intensities + standard deviation) and used to calculate the frequency of positive nuclei per core. The average of tumor cores from the same patient was used for analysis. The intra class correlation (ICC) between the duplicate cores was 0.68 and 0.70 (*p* < 0.001, Spearman) for nuclear p65 and nuclear RelB respectively.

### Statistical analyses

Statistical analyses were performed with SPSS software 16.0 (SPSS Inc. Chicago, IL, USA). A comparison between normal adjacent and matched tumor cores was assessed using a non-parametric Wilcoxon test. The correlation with clinicopathological variables was estimated with a non-parametric Spearman correlation test. Due to the inherent nature of immunofluorescence, the cores were considered positive for nuclear NF-κB when values were at least 5% over background staining. We used Receiver Operative Characteristic (ROC) curves calculation from the frequency of all nuclei to determine the threshold value for each NF-κB subunit in Kaplan-Meier analysis. BCR-free survival curves were plotted using the Kaplan–Meier estimator and the log-rank test was used to evaluate significant differences. The ratio of p65/RelB was calculated from the frequency of nuclear p65 as compared to nuclear RelB. The univariate and multivariate proportional hazard models (Cox regression) were used to estimate the hazard ratios for each NF-κB as categorical variable, while missing values were not considered. Multivariate analysis was performed using an Enter stepwise hazard model on univariate analysis that were required for entry into the model. A sample size of n = 10k was considered before applying the multivariate model (n = number of events, k = number of variables). The covariables were not time dependent. Additional clinicopathological variables included pre-operatory PSA, Gleason score, surgical margin status, extra-prostatic extension and seminal vesicle involvement.

### Ethics Statement

An Ethical approval for the study was obtained from the appropriate review board (Comité d’éthique de la recherche du CHUM). A written informed consent was obtained from all participants.

## Results

### Immunofluorescent multi-staining analysis of p65 and RelB

The aim of the study is to analyze p65 and RelB in normal adjacent or malignant epithelial prostate cancer tissues on the same TMA slide using a quantitative and semi-automated process. A quantitative analysis provides continuous data, allowing for a wider range of detection and more accurate determination of low and high intensity staining. In order to facilitate this process and achieve maximal specificity, we chose to implement a multi-immunofluorescent staining technique (IF).

First, we selected epithelial areas to be analyzed. Because of its known constitutive expression in prostatic epithelial cells, cytokeratin 18 (CK18) was chosen to identify epithelial cells in our specimens [37]. However, we observed that tissue samples stained by IHC produced variable CK18 expression levels and that staining was weaker in poorly differentiated tumors (data not shown), thus reducing the accuracy of tumor cell identification in advanced prostate cancer tissues. To overcome this, we selected CK19 and PSA as epithelial antigens, since they are known to be highly expressed in prostatic epithelial cells [37, 38]. Visual verification of the simultaneous staining of CK18, CK19 and PSA with orange fluorescent dyes (A546 for CK18 and CK19, and Cy3 for PSA) confirmed full epithelial coverage by this tri-antigen staining cocktail, providing the sensitivity and specificity required to identify prostatic epithelial cells regardless of the level of tissue differentiation (S1 Fig).

**Fig 1:**
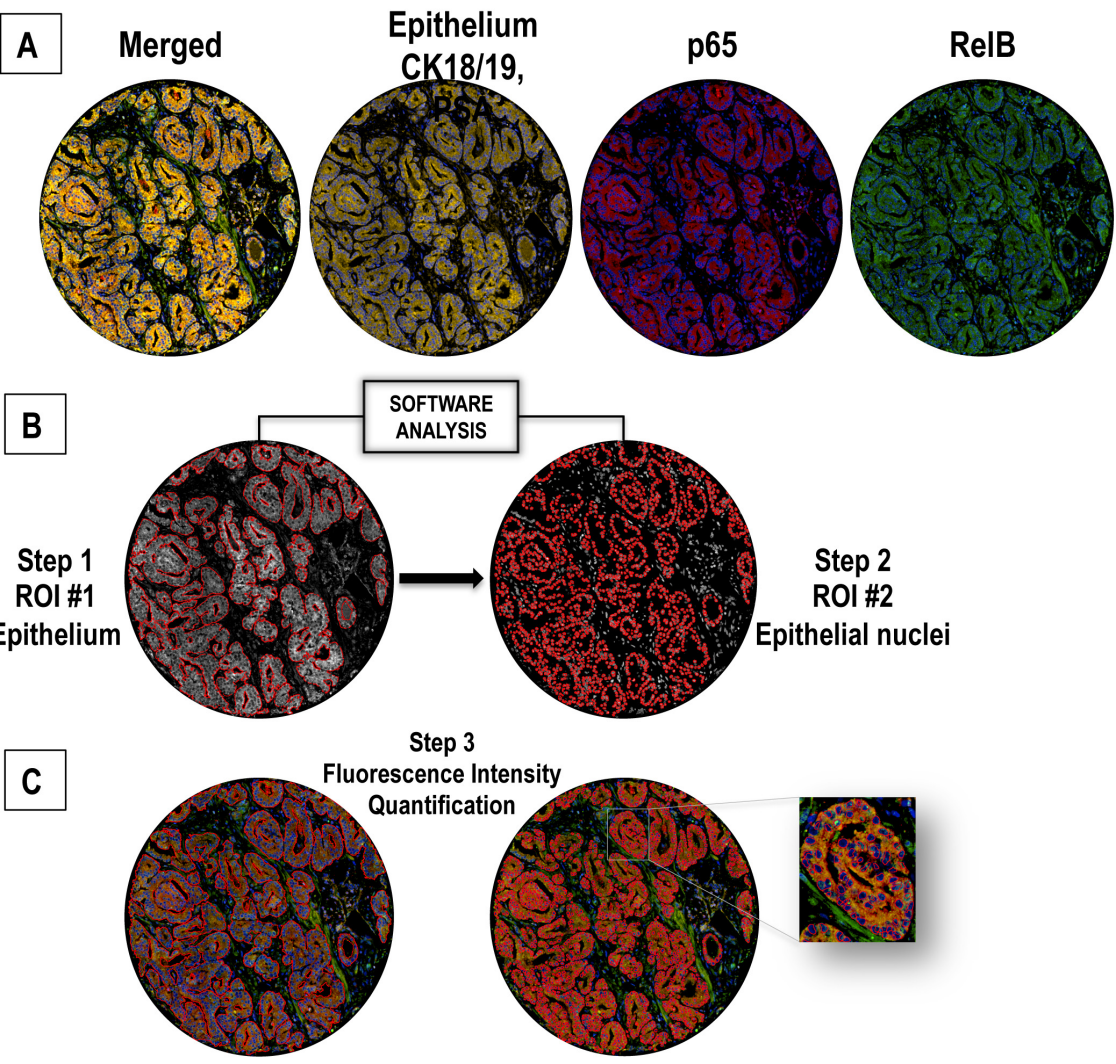
Nuclear co-localization of p65 and RelB by a quadruple multiple staining approach. **A.** Epithelial staining with CK18, CK19 and PSA defines the epithelial mask using orange fluorochromes (A546, Cy3). **B.** Identification of epithelial area (presented in grey for optimal contrast in subsequent analyses) as ROI #1 (region of interest #1). Identification of nuclei (grey surrounded by red tracing) as ROI#2 from pre-defined ROI#1. P65 and RelB fluorescence were subsequently evaluated separately in ROI#1 and #2. **C.** RelB staining with green fluorescent dye (A488) and p65 staining with red fluorescent dye (Cy5). Steps 1, 2 and 3 correspond to the fluorescence analysis process followed using Visiomorph DP software.

The orange epithelial mask was used with the DAPI nuclear staining to restrict quantification of p65 and RelB to epithelial nuclei. The subunits p65 and RelB were stained with Cy5 (red) and A488 (green) dyes, respectively (Fig1). After this quadruple IF staining, we performed a quantitative analysis of p65 and RelB from scanned images using the immunofluorescence image analysis program VisiomorphDP. A threshold value of fluorescence intensity was defined to discriminate positive and negative fluorescent signals, which was used to determine the specific frequency (%) of p65- or RelB- positive nuclei in each tissue core (including double positive nuclei) (Fig 1).

### Comparison of p65 expression using immunohistochemistry and quantitative automated immunofluorescence analysis

**Fig 2:**
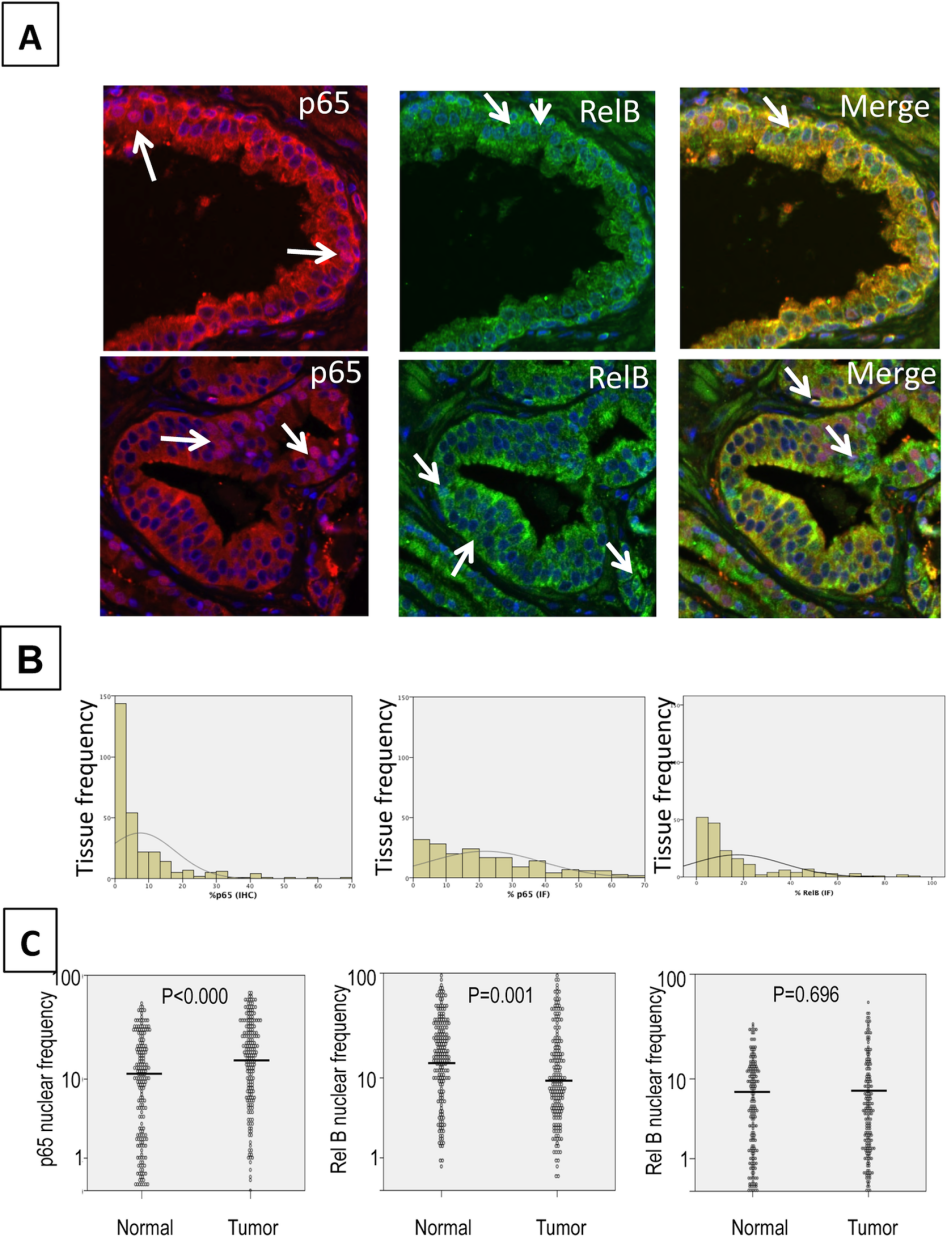
Example of immunofluorescence of p65 and RelB. **A.** Magnification (40X) highlighting glandular structures in prostate cancer tissues. Merge: superimposed images of RelB (A488) in green, p65 (Cy5) in red and nuclei (DAPI) in blue, in normal adjacent (top) or cancer tissues (bottom). Arrows show stained nuclei. **B**. Comparative quantification from IHC (left) and IF (right) staining of p65. Graphs show the frequency distribution of nuclear p65 as evaluated visually (IHC) or automatically (IF). **C**. Frequency of nuclear p65 (left), RelB (middle) and double stained nuclei (right) in normal adjacent and tumor tissues. The comparison between adjacent normal and tumor cores was conducted using a Wilcoxon‘s test.

TMAs, containing patient-matched cores from 200 prostatic tumor tissues and normal adjacent epithelium were stained, scanned and evaluated for p65 and RelB positive signal. After revision of clinical data only 11 cases were excluded as they did not meet the inclusion criteria anymore. Presence of p65 and RelB was mostly observed in epithelium, with both cytoplasmic and nuclear compartments exhibiting positive staining (Fig 2A). Using the automated scoring program, we were able to identify negative, single p65 (Cy5) or RelB (A488), and double (Cy5/A488) stained nuclei (Fig 2A).

To evaluate the performance of this IF technique, implemented for quantification of nuclear markers in prostate cancer tissue, we compared quantification results from the automated analysis with results obtained using traditional IHC staining. In both cases, the frequency of p65 stained nuclei was evaluated. We obtained a significant correlation between both datasets (r = 0.410, *p* < 0.001 Spearman), which is in the same range as the correlation between duplicate cores within the given technique. The traditional IHC method, based on visual scoring, was unable to distinguish between low (or no) staining, thereby leading to an overestimation of negative p65 expression cores (Fig 2B). Using the Kaplan-Meier estimation curves, we also compared the survival association between nuclear p65 frequency and risk of BCR, as previously analyzed in numerous other prostate cancer cohorts [21, 22, 25, 39]. Nuclear p65 was found to be similarly and significantly associated with risk of BCR in both the traditional IHC and software-analyzed IF samples (Log rank = 4.38, *p* = 0.036 and Log rank = 4.83, *p* = 0.0028, respectively). Altogether, these results validate the use of automated analysis by IF for the evaluation of NF-κB subunit localization and quantification in prostate cancer tissues.

### Analysis of nuclear distribution of p65 and RelB in prostate cancer tissues

While p65 nuclear localization was increased in prostate tumors compared to normal adjacent tissues (Fig 2C, *p* < 0.000, Wilcoxon test), RelB exhibited a higher expression in normal adjacent tissue vs. tumor tissue (p = 0.001, Wilcoxon test, Fig 2C). The percentage of double stained p65/RelB nuclei did not show any significant variation between normal and tumor tissues (Fig 2C).

In tumor cores, the activation of the classical pathway, as seen by nuclear p65, was significantly correlated with the activation of the alternative pathway, as represented by nuclear RelB localization (r = 0.522, p < 0.001 Spearman). To estimate the amount of interaction between the two NF-κB pathways, we compared the nuclear frequency of each subunit in the presence or absence of the other subunit. When nuclear RelB was expressed, there was a concomitant increase in p65 nuclear localization frequency compared to cores without nuclear RelB (27% and 19%, respectively). Similarly, the presence of nuclear p65 was associated with an increase in nuclear localization of RelB (18% and 10%, respectively).

### Correlation of NF-κB variables with clinico-pathological parameters

Next we evaluated the correlation between nuclear p65 (representing activation of the classical pathway) or RelB (representing activation of the alternative pathway) and clinicopathological parameters. Although nuclear expression of these two proteins was not significantly correlated with most prostate cancer aggressiveness parameters, there was a significant correlation between nuclear p65 and seminal vesicle involvement (r = 0.121, p = 0.048, Spearman, Table 1).Furthermore, there was a trend between nuclear RelB and patient Gleason score (r = -0.105, p = 0.081, Spearman, Table 1).

**Table 1:** Spearman correlation test between NF-κB and clinic-pathological parameters.

	Nuclear frequency of p65	Nuclear frequency of RelB
Gleason	r = 0.014 (p = 0.812)	r = -0.105 (p = 0.081)
Lymph node invasion	r = 0.072 (p = 0.302)	r = 0.048 (p = 0.489)
Seminal vesicule involvement	**r = 0.121 (p = 0.048)**	r = 0.011 (p = 0.861)
Extraprostatic extension	r = 0.038 (p = 0.538)	r = 0.089 (p = 0.153)
Surgical margin	r = 0.04 (p = 0.514)	r = 0.048 (p = 0.437)
APS pre-op	r = 0.014 (p = 0.812)	r = 0.047 (p = 0.442)
Stage	r = 0.079 (p = 0.281)	r = 0.045 (p = 0.535)

Significant Spearman correlations are indicated in bold. Spearman correlations were considered significant at *p* < 0.05.

### Analysis of the co-expression of p65 and RelB

**Fig 3:**
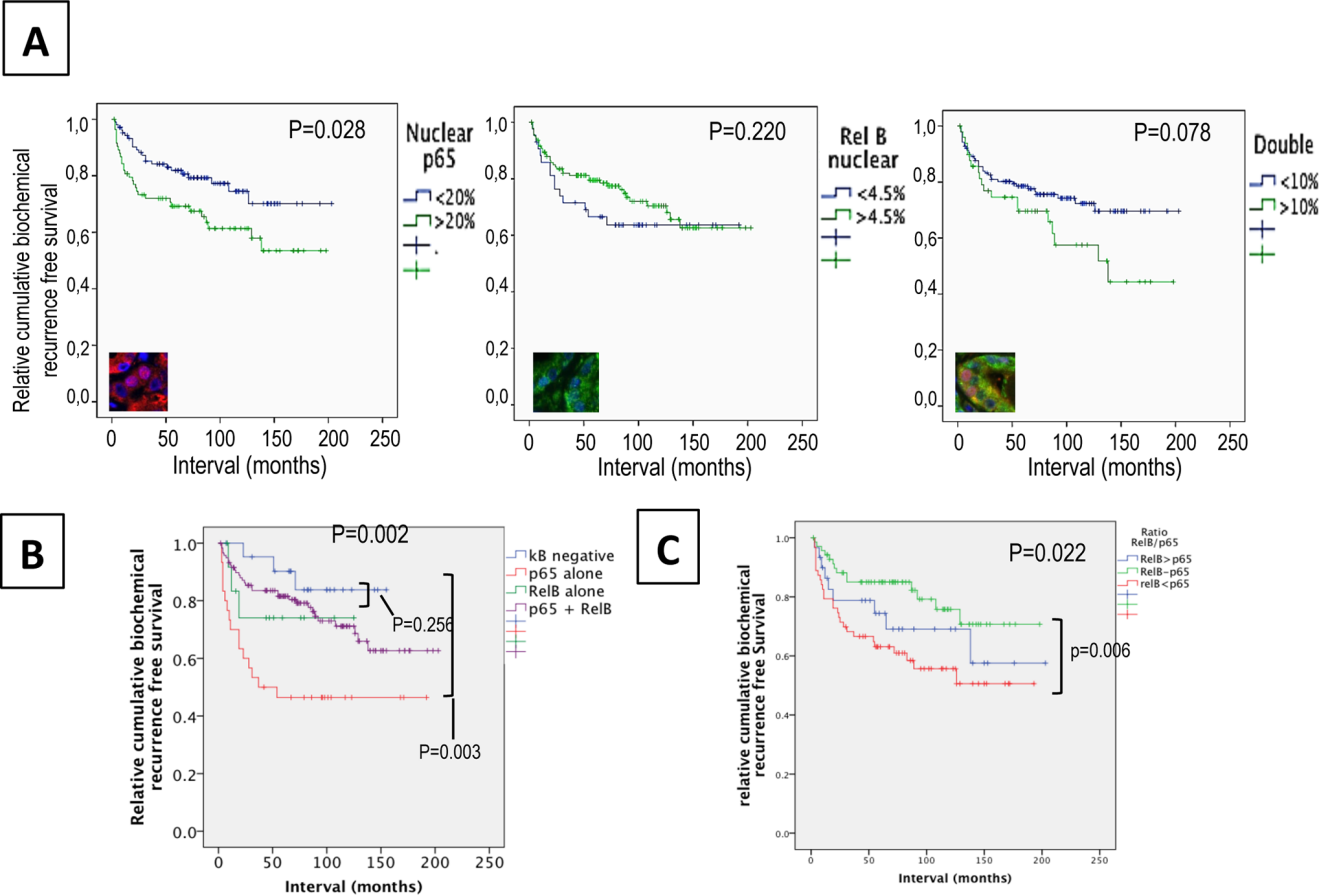
Association between nuclear p65 and/or RelB and biochemical recurrence in prostate cancerpatients. Kaplan–Meier biochemical recurrence-free survival curves **A.** High (>20%) and low (<20%) frequency of nuclear p65 in the epithelia of cancer tissues. **B.** High (>5%) and low (<5%) frequency of nuclear RelB in the epithelia of cancer tissues. Significance (*p*) is indicated by log rank. **C.** Double nuclear epithelial staining of p65 and RelB. **D.** Epithelial and nuclear staining of p65 and RelB. The κB negative variable represents patients without positive tissue cores for p65 and RelB. The variable p65 alone or RelB alone represents patients positive for only nuclear p65 or nuclear RelB. The variable p65 + RelB are patients with presence of both nuclear subunits in the same core. **E.** Analysis of the epithelial proportion of nuclear p65 to RelB in tissue cores within both p65 and RelB staining. The ratio p65/RelB represents the frequency of p65 to the frequency of RelB. The variable p65 > RelB represents a ratio of at least 2 fold more p65 than RelB; p65-RelB represents a ratio between 0.50 and 2 fold, and RelB>p65 represents a ratio of at least 2 fold more RelB than p65.

To assess the role of each NF-κB pathway in prostate cancer progression and the potential crosstalk between both pathways, we used biochemical recurrence (BCR) as a functional indicator of these activities. The nuclear frequency of p65 was associated with overall BCR (*p* = 0.028, Log rank = 4.843, Fig 3A). A Cox regression analyses confirmed the association between p65 and BCR in univariate (HR = 1.93, *p* = 0.017) and multivariate models (HR = 3.15, *p* = 0.003), including clinicopathological parameters such as Gleason score, extra-prostatic extension, lymph node invasion, seminal vesicle involvement, and surgical margins (Table 2). In contrast to p65, Kaplan-Meier estimation and univariate Cox regression analyses showed that the RelB status alone was not associated with BCR (*p* = 0.301, Fig 3B and Table 2). Surprisingly, the frequency of p65/RelB double positive nuclei was not significantly predictive of BCR but a trend towards a worse prognosis was observed (*p* = 0.078, Fig 3C).

**Table 2:** Univariate and multivariate Cox regression analysis.

	Univariate		Multivariate		Multivariate	
	p	HR (95% CI)	p	HR (95% CI)	p	HR (95% CI)
Nuclear p65	0.017	1.93 (1.22–3.33)	0.004	2.30 (1.31–4.04)	-	-
Nuclear RelB	0.301	0.73 (0.401–1.33)	-	-	0.162	0.65(0.35–1.19)
Gleason	0.000	2.10 (1.57–2.81)	0.004	1.57 (1.15–2.13)	0.009	1.51 (1.11–2.08)
Surgical margin	0.000	4.07 (2.33–7.12)	0.002	2.591 (1.41–4.77)	0.002	2.61 (1.41–4.84)
Pre-op PSA	0.000	1.089 (1.042–1.139)	0.112	1.044(0.99–1.10)	0.024	1.06 (1.01–1.12)
Stage	0.000	4.54(2.65–7.78)	0.003	2.501(1.36–4.62)	0.005	2.83(1.29–4.39)

Cox regression models: significance (p < 0.05) and Hazard ratio (HR) are indicated. CI: Confidence interval. PSA: Prostate Specific Antigen. NF-κB variables (p65 and RelB): status in cancer tissues. Significance is considered at p < 0.5.

The activity and interaction between the p65 and RelB pathways can vary widely within a single tissue which can contains a mixed pattern of expression with cells showing only p65 or only RelB while others being double positive. To determine the individual roles of p65 and RelB, we compared patient tissues with activation of a single pathway, represented by nuclear frequency of either p65 or RelB, and patients with no NF-κB activity (Fig 3D). Patients with only p65 activity showed a significantly shorter time of recurrence as compared to patients with no NF-κB activity (138 months and 98 months respectively, Log rank = 8.8, *p* = 0.002,) or compared to patients with RelB only (*p* = 0.021, Log Rank = 5.4, Fig 3D). This confirms that the classical NF-κB pathway strongly promotes the progression of prostate cancer. Conversely, in our patient cohort, RelB activity alone was not found to affect recurrence when compared to patients without NF-κB activation *(p* = 0.741, Fig 3D). Interestingly, the nuclear presence of both p65 and RelB in the same core was not significantly associated with BCR (*p* = 0.252), suggesting that RelB may counteract the biological effect of p65.

To define the potential of the role of RelB on p65 activity, we investigated the impact of each pathway on the other in varying proportions. The ratio of nuclear p65 and RelB was used to segregate patient tissues with double staining of p65 and RelB (Fig 3E). Three categories were made: patients with a higher proportion of nuclear p65 than RelB (greater than 2 fold), patients with a higher proportion of nuclear RelB than p65, and patients with relatively equal numbers of p65- and RelB-positive nuclei (up to 2 fold difference). As expected, a high level of nuclear p65 was associated with a significant risk of BCR (Log rank = 6.8, *p* = 0.026 Fig 3E) compared to cases similar level of p65 and RelB. However, when nuclear RelB was also present, the p65-dependent increase in risk was abolished (*p* = 0.344), supporting the hypothesis that RelB interferes with the cancer-related biological activity of p65.

## Discussion

One of the multiple advantages of a semi-automated approach to antigen scoring is the ability to generate of robust and continuous data allowing for a more precise quantification of biomarker expression as compared to the traditional categorical IHC scoring. Comparison of IHC and IF data showed superior sensitivity and accuracy for the latter in the quantification of low frequency cores. In addition, we used the Kaplan-Meier estimator to compare the efficiency of the two methods in the evaluation of risk association. While IF staining and image analysis was a slightly better discriminator of recurrence risk between the two groups compared to IHC, the difference in accuracy was minimal (Log rank = 4.38, *p* = 0.036 and Log rank = 4.83, *p* = 0.0028, respectively, S2 Fig) and did not overwhelmingly favor the use of IF for this purpose. In addition we found that IF approach is time consuming and expensive compare to the standard IHC approach. It should be noted that an evaluation of the automated approach for sensitivity, efficiency and reproducibility and experimental variability (including intra- and inter-assay assay) within whole tissue sections would also have to be performed before any consideration is given to applications in clinical settings. However, the consistency of the comparison, lends confidence to an automated analysis approach to evaluate the role of NF-κB in prostate cancer progression.

An advantage of the IF technique is the ability to stain multiple proteins on a single tissue slide. Coupled with an automated detection system, IF multi-staining allows for the accurate detection and distinction of several fluorochromes that would otherwise not be discernable by the human eye. However it allows using continuous quantification of biomarker which give a better representation of their biological expression. Here, we were able to detect the co-localization of p65 and RelB specifically in epithelial cell nuclei by combining Cy5 dye and Alexa Fluor 488. Since prostate cancer tissue is heterogeneous and contains differing amounts of stromal tissue, we combined the detection of NF-κB subunits with antibodies against structural markers of epithelium coupled to Cy3 and Alexa Fluor 546 dyes in order to restrict quantification to the epithelial compartment. In our IF analysis we could not account for the presence of benign glands in tissue cores due to technical limitations based on the number of simoutaneous fluorochromes we could use. This is the main limitation to evaluation of NF-κB subunits as potential clinical prognostic biomarkers in prostate tissues in this work.

While immunofluorescence is now well developed and is often used in translational research, particularly for FISH assays, there are still some drawbacks to consider before applying any immunofluorescence data in the clinical setting. First, auto-fluorescence is a major problem for background staining which is difficult to eliminate in the data analysis. Here auto-fluorescence has been here reduced by the use of Sudan black but for translational research purposes, confocal laser scanning microscopy should be used to optimize the correction for auto-fluorescence and background [40]. IF also encounters problem of signal quenching at excitation particularly when large slides are used and scanning times are long as seen on TMA. A further point to consider is the reproducibility of results and the need to create a standard threshold signal that defines a clinically relevant cut-off. This has not been evaluated in this study but needs to be defined if p65 and RelB are to be used as biomarkers in prostate cancer.

Unfortunately, in our study, due to a limitation of the software we were not able to quantify the intensity of p65 and RelB individually in the same nuclei on a large scale. We had to estimate the global staining of the all nuclei in the same core and this does not account for the potential tumor heterogeneity.

The implication of the classical NF-κB pathway in prostate cancer progression has been extensively investigated (reviewed in [20]). In these studies, it was shown that the frequency of p65 nuclear distribution, as evaluated by IHC in prostatic tumor cells, was correlated with either Gleason score, presence of lymph node metastasis, seminal vesicle invasion or with surgical margins [8, 24, 25, 39, 41]. In addition, it was demonstrated that the presence of nuclear p65 in prostate cancer tissues predicted BCR, suggesting that the classical NF-κB pathway is indeed a central player in prostate cancer oncogenesis [25, 39, 42]. Therefore, in using an alternative IF-based approach, the current study further confirms the association between the activation of the classical NF-κB pathway, as estimated by p65 nuclear localization, and the aggressiveness of the disease (Tables 1 and 2).

Several lines of evidence suggest that the alternative NF-κB pathway is also involved in cancer progression and in bone metastasis. *In vitro* breast cancer studies associate RelB with proliferation, migration and invasion of the disease [43, 44], while in a fibroblast-based model, RelB has an anti-proliferative role via p53 activation [45]. A pro-apoptotic function for RelB has also been reported in renal epithelial cells [46]. More interestingly, RankL, a cytokine that mediates differentiation of macrophages into osteoclasts, was proven to induce activation of both p65 and RelB, which may favor bone metastasis in prostate cancer [47]. It has also been reported that RelB overexpression in the LnCaP prostatic cell line, whose the basal classical NF-κB activity is moderate, promotes the in vivo tumor growth into murine model and increases the ability to form colonies in soft agar [48]. However, in the 22Rv1 prostatic cell line that is exempt of any classical NF-κB activity, the alternative NF-κB pathway activation triggers a stress-induced autophagy and tends to delay the *in vivo* tumor growth in the corresponding xenograft murine model. These studies could illustrate the complex link between both the classical and alternative NF-κB pathways in prostate cancer biology [26]. While the p65 classical NF-κB subunit is clearly associated with oncogenic properties, the role of RelB remains to be fully elucidated. Altogether, it appears that RelB has a complex role that may depend on the cell types and concomitant stimuli.

In contrast to p65, we did not observe any strong associations between nuclear RelB and disease progression. Nuclear localization of RelB was not associated with an overall risk of BCR (Fig 3B) in our cohort, though there was a trend towards lower risk of BCR before 5 yrs (*p* = 0.067 log Rank) and lower Gleason score (*p* = 0.081). To our knowledge, there are few IHC studies assessing the role of RelB in cancer. In ovarian cancer, a lack of association of RelB expression and patient prognosis was reported [49]. Similarly, nuclear RelB in retinoblastoma tissue was not associated with any pathological parameters [50]. Altogether, these studies do not identify RelB alone as a potential prognostic marker. The combination of RelB to RelA showed a slightly increased accuracy in discriminating early recurrence cases (Fig 3D) but further investigations in an appropriate translational setting are needed to address the potential of RelB and p65 before consideration of their application in clinical decision. While the clinical potential of RelB seems low, this does not preclude a functional role for this alternative NF-κB pathway in cancer or even as a therapeutic target. Indeed, we did observe that RelB activity in prostate cancer tissues decreased the risk of recurrence associated with p65 nuclear localization, suggesting that RelB may interfere with the pro-tumoral function of the classical pathway. In our cohort, the number of patients was relatively limited and the role of RelB and p65 should, in future work, be estimated on a larger scale.

Several levels of cross-talk could exist between the classical and alternative pathways. At the signaling level, an antagonistic interaction can exist, in which the activation of one pathway negatively impacts the other pathway. However, we observed a direct correlation between nuclear p65 and RelB (0.522, p < 0.001), where nuclear translocation of one subunit was increased when the other pathway was also activated. Another level of interference is an indirect crosstalk via gene induction, such as induction of p52 or repression of target genes induced by the classical pathway [51]. It is reasonable to suggest that such a mechanism could exist in prostate cancer, since the risk association of p65 was lost in double positive nuclei unless the frequency of p65 was at least twice as high as that of RelB (Fig 3). Furthermore, a direct interaction between RelB and p65 has also been reported. Association of p65 with RelB may mediate suppression of the latter’s DNA binding on target genes [52]. Interestingly, we have previously observed a binding between p65 and RelB in the 22Rv1 cell line, supporting the possibility of a direct competition between p65 and RelB in prostate cancer tissue[26]. An indirect cross talk could also exist through the induction of protein expression acting in a paracrine manner as an environmental factor. The precise mechanism by which RelB could exert its suppression on p65 in prostate cancer cells still remains to be elucidated, and additional studies are required to further investigate the underlying role of RelB and the alternative pathway in prostate cancer.

## Supporting Information

S1 Fig Definition of epithelial mask. **A.** Immunohistochemistry illustrating a differential expression of CK18, CK19 and PSA in normal prostate tissue and tumor tissues cores from patients with either low or high grade disease. **B.** Simultaneous immunofluorescence staining with CK18, CK19 and PSA in normal prostate tissue and tumor tissues cores from patients with either low or high grade disease. Secondary antibodies were conjugated with A546 (CK18 and CK19) or Cy3 (PSA), each emitting fluorescence recognized in the orange range. All images at a 10X magnification. CK: cytokeratin, PSA: Prostate specific antigen.
(TIF)

S2 Fig Comparative association of nuclear p65 and disease recurrence analyzed by immunofluorescence and immunohistochemistry. Kaplan–Meier biochemical recurrence-free survival curves in patients with prostate cancer **A.** High (>20%) and low (<20%) frequency of nuclear p65 detected by immunofluorescence. **B.** High (>2.5%) and low (<2.5%) frequency of nuclear p65 detected by immunohistochemistry. Significance (*p*) is indicated by log rank.
(PNG)

S1 Table Tissue microarray data. (XLSX)
