# Supplementary figures and images for: Potential Cross-Talk between Alternative and Classical NF-κB Pathways in Prostate Cancer Tissues as Measured by a Multi-Staining Immunofluorescence Co-Localization Assay
Source: PLoS One. 2015 Jul 17;10(7):e0131024. doi: 10.1371/journal.pone.0131024 (PMC4505937; doi:10.1371/journal.pone.0131024)

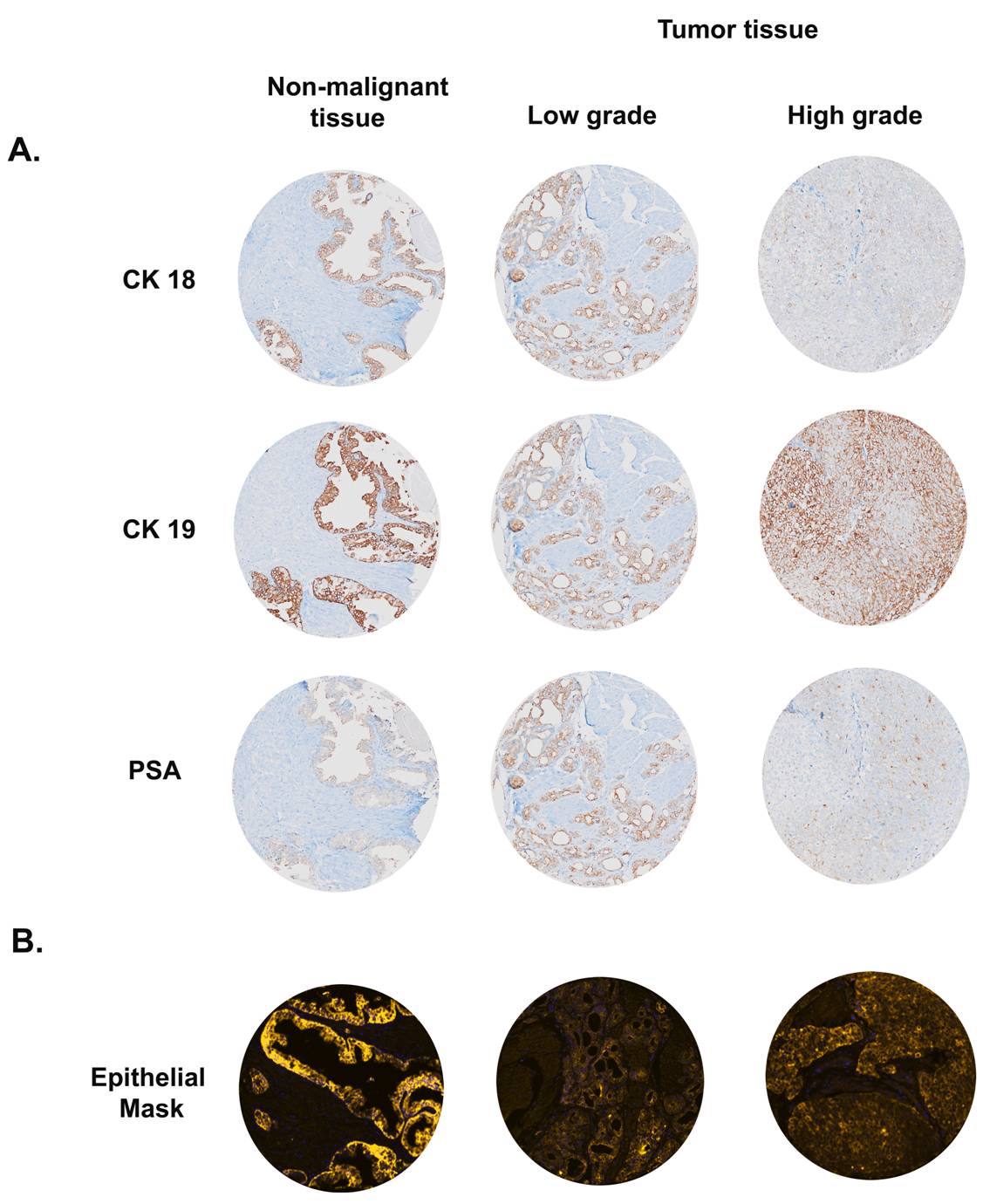

Supplement: S1 Fig — A. Immunohistochemistry illustrating a differential expression of CK18, CK19 and PSA in normal prostate tissue and tumor tissues cores from patients with either low or high grade disease. B. Simultaneous immunofluorescence staining with CK18, CK19 and PSA in normal prostate tissue and tumor tissues cores from patients with either low or high grade disease. Secondary antibodies were conjugated with A546 (CK18 and CK19) or Cy3 (PSA), each emitting fluorescence recognized in the orange range. All images at a 10X magnification. CK: cytokeratin, PSA: Prostate specific antigen. (TIF) [file pone.0131024.s001.tif]

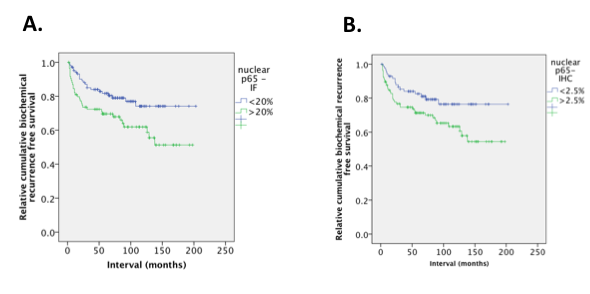

Supplement: S2 Fig — Kaplan–Meier biochemical recurrence-free survival curves in patients with prostate cancer A. High (>20%) and low (<20%) frequency of nuclear p65 detected by immunofluorescence. B. High (>2.5%) and low (<2.5%) frequency of nuclear p65 detected by immunohistochemistry. Significance (p) is indicated by log rank. (PNG) [file pone.0131024.s002.png]
